# The Dual Benefit of Plant Essential Oils against *Tuta absoluta*

**DOI:** 10.3390/plants12050985

**Published:** 2023-02-21

**Authors:** Saoussen Ben Abdallah, Chaymaa Riahi, Sandra Vacas, Vicente Navarro-Llopis, Alberto Urbaneja, Meritxell Pérez-Hedo

**Affiliations:** 1 Instituto Valenciano de Investigaciones Agrarias (IVIA), Centro de Protección Vegetal y Biotecnología, CV-315, Km 10.7, 46113 Moncada, Valencia, Spain; 2 Horticultural Science Department, Southwest Florida Research and Education Center, University of Florida/IFAS, Immokalee, FL 34142, USA; 3 Centro de Ecología Química Agrícola, Instituto Agroforestal del Mediterráneo, Universitat Politècnica de València, Camino de Vera s/n, 46022 Valencia, Valencia, Spain

**Keywords:** *Nesidiocoris tenuis*, yarrow, garlic, rosemary, marigold, thyme, *Solanum lycopersicum*, plant defenses

## Abstract

Plant essential oils (PEOs) are being studied as a potential alternative to synthetic pesticides in agriculture. PEOs have the potential to control pests both directly, by being toxic or repellent to pests, and indirectly, by activating plant’s defense mechanisms. In this study, the effectiveness of five PEOs (*Achillea millefolium*, *Allium sativum*, *Rosmarinus officinallis*, *Tagetes minuta*, and *Thymus zygis*) on controlling *Tuta absoluta* and their impact on the predator *Nesidiocoris tenuis* was examined. The study revelead that PEOs from *A. millefolium* and *A. sativum*-sprayed plants significantly reduced the number of *T. absoluta*-infested leaflets and did not affect the establishment and reproduction of *N. tenuis*. Additionally, the spraying of *A. millefolium* and *A. sativum* increased the expression of defense genes in the plants, triggering the release of herbivory-induced plant volatiles (HIPVs), such as C6 green leaf volatiles, monoterpenes, and aldehydes, which can be messengers in tritrophic interactions. The results suggest that PEOs from *A. millefolium* and *A. sativum* can provide a dual benefit for controlling arthropod pests, as they can directly exhibit toxicity against these pests while also activating plant defense mechanisms. Overall, this study provides new insights into using PEOs as a sustainable solution for controlling pests and diseases in agriculture, by reducing synthetic pesticides and promoting the use of natural predators.

## 1. Introduction

The use of conventional chemical pesticides in agriculture has led to the development of cross-resistance in insect populations and potential risks and hazards for the environment and non-target organisms [1]. To address these concerns, there is a push to reduce hazardous chemical pesticides and increase the use of eco-friendly products through Integrated Pest Management (IPM) programs [2,3]. This shift towards sustainable food production is necessary to ensure food safety and address worldwide environmental concerns.

Integrated Pest Management (IPM) often includes the use of Plant Essential Oils (PEOs) as a method of controlling pests [4]. PEOs are mixtures of secondary metabolites that are derived from aromatic and medicinal plants. They are a sustainable alternative to chemical pesticides, as they have bio-insecticide properties and little negative environmental impact [5]. The use of PEOs as part of an IPM program is seen as an eco-friendly option for pest control and consumer products.

PEOs have been shown to have repellent, insecticidal, and growth-reducing effects on a variety of insects [6]. This is due to their chemical composition, which can include terpene hydrocarbons such as monoterpenes and sesquiterpenes, as well as oxygenated compounds like phenols, alcohols, aldehydes, and esters. These compounds affect the preference and performance of pests towards their host plants, by influencing factors such as oviposition, feeding behavior, growth rate, development, and reproductive success [7]. The toxic and behavioral effects observed in insects are primarily attributed to the presence of monoterpenoids and sesquiterpenoids in the composition of PEOs [8,9].

PEOs have been found to have indirect plant protection properties in addition to their direct insecticidal properties. These properties involve modulating the plant’s metabolic network related to phenolic compounds synthesis, defense-related enzymes, and the emission of herbivore-induced plant volatiles (HIPVs) [6,7,10]. This opens new possibilities for IPM prospects. PEOs not only protect the plant directly by affecting the pest’s preference and performance, but also by strengthening the plant’s defense mechanisms [6,10].

HIPVs are a key aspect of indirect plant protection. They are emitted by plants when they are under attack by pests and/or the presence of zoophytophagous predators [11,12,13,14]. HIPVs are known to defend plants by repelling, deterring, and being toxic to the pests, as well as by attracting the natural enemies of herbivores [7,11]. This helps to protect the plant from damage. HIPVs typically comprise monoterpenes, sesquiterpenes, and green leaf volatiles (GLVs) [10]. The production of HIPVs is regulated by phytohormone pathways such as jasmonic acid (JA), salicylic acid (SA), ethylene, and abscisic acid (ABA). These pathways are activated in response to herbivore attacks and are key regulators of plant defense responses.

Previous research has demonstrated the potential role of PEOs, including garlic (*Allium sativum*, Amaryllidaceae), rosemary (*Rosmarinus officinalis*, Lamiaceae), absinthe (*Artemisia absinthium*, Asteraceae) and oregano (*Origanum vulgare*, Lamiaceae), in acquired defense responses against pathogens and herbivory attacks [6]. PEOs can modulate the expression of phytohormone and plant metabolic pathways, triggering the production of HIPVs through the involvement of JA, SA and ABA pathways [6]. However, more research is needed to determine the specific responses of plants to PEOs and pest attacks [15]. The application of PEOs to control insects at the biochemical and/or transcriptomic level is encouraged, as it can trigger the production of HIPVs.

The South American tomato pinworm, *Tuta absoluta* (Meyrick) (Lepidoptera: Gelechiidae), is a highly invasive pest that damages tomato crops in both open-field and greenhouse cultivation [16,17]. It has rapidly expanded from Central America to Africa and has become a globally and economically important pest due to its short generation period and ability to develop insecticide resistance [16,18,19]. The development and implementation of IPM tools have been extensively researched to achieve successful and sustainable management of *T. absoluta* worldwide [16,17,19,20,21,22].

Many PEOs extracted from aromatic and medicinal plants have been found to have significant insecticidal toxicity towards *T. absoluta* eggs and larvae, mortality, and repellence under laboratory conditions, suggesting their potential use in IPM programs [23,24,25,26,27,28]. The garlic PEO has been found to be particularly effective in controlling *T. absoluta*, while having low mortality towards its generalist predator *Nesidiocoris tenuis* Reuter (Hemiptera: Miridae) and no phytotoxic effects on tomato plants [25]. However, there is limited field evidence on using PEOs as elicitors on tomato plants against *T. absoluta* and its predator *N. tenuis*.

In this study, we aimed to investigate the effectiveness of five PEOs [yarrow (*Achillea millefolium*, Asteraceae), garlic (*A. sativum*), rosemary (*R. officinalis*), marigold (*Tagetes minuta*, Asteraceae), and thyme (*Thymus zygis*, Lamiaceae)] on controlling *T. absoluta* and their side-effects on the performance of the predator *N. tenuis*. In addition, using a Y-tube olfactometer, we studied the response of *T. absoluta* and *N. tenuis* to tomato plants that had been previously sprayed with these PEOs and tomato plants that had not. We also determined the expression of defensive genes [proteinase Inhibitor II (*PIN2*), abscisic acid stress ripening protein (*ASR1*) and pathogenesis-related protein precursor (*PR1*)] and the emission of HIPVs from tomato plants that had been primed with *A. millefolium* and *A. sativum* oils by using real-time polymerase chain reaction (RT-PCR) and headspace solid-phase microextraction (HS-SPME) coupled with gas chromatography/mass spectrometry (GC-MS). The results obtained in this study offer significant and strong potential for developing sustainable control of *T. absoluta* in tomato crops treated with *A. millefolium* or *A. sativum* PEOs.

## 2. Results

### 2.1. Olfactory Response of Tuta absoluta and Nesidiocoris tenuis to PEOs

**Figure 1:**
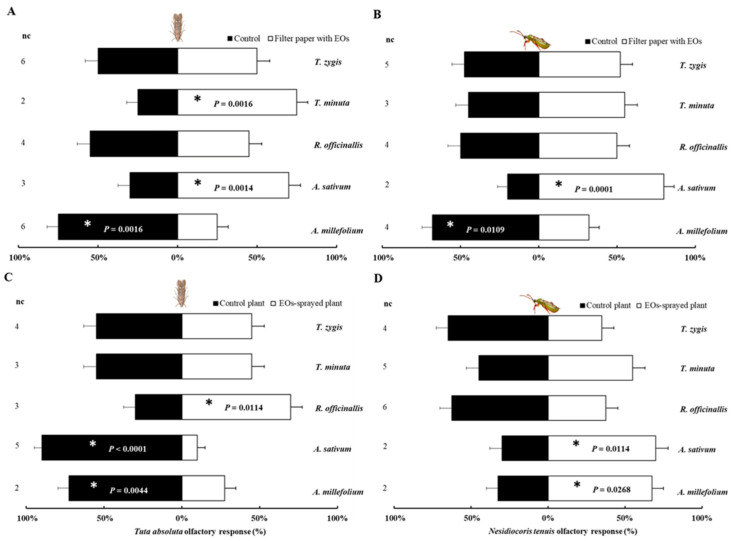
Response of the herbivore *Tuta absoluta* and the natural enemy *Nesidiocoris tenuis* females to plant essential oils, using a Y-tube olfactometer. (**A**,**B**) *T. absoluta* and *N. tenuis* were exposed to filter paper sprayed or not with PEOs. (**C**,**D**) *T. absoluta* and *N. tenuis* response to tomato plants previously sprayed with PEOs. The means of 40 repetitions in each treatment ± SE. nc (no choice) is the number of individuals that did not make a choice. Asterisks indicate significant differences in the distribution of side-arm choices (*χ*^2^ tests; *p* < 0.05).

The response of *T. absoluta* and its predator *N. tenuis* in a Y-tube olfactometer when exposed to five PEOs and a control was investigated, and the results are presented in Figure 1. *Tuta absoluta* displayed a preference for the control over the odor source containing *A. millefolium* oil (*χ*^2^ = 10, *p* = 0.0016), but preferred the glass chamber containing *A. sativum* and *T. minuta* (*χ*^2^ = 6.4, *p* = 0.0114 and *χ*^2^ = 10, *p* = 0.0016, respectively) (Figure 1A). Similarly, the predator *N. tenuis* preferred the control (*χ*^2^ = 6.48, *p* = 0.0109) over *A. millefolium* oil but was significantly attracted to the odor of *A. sativum* (*χ*^2^ = 14.4, *p* = 0.0001) *(*Figure 1B).

On the other hand, plants sprayed with *A. millefolium* and *A. sativum* oils exhibited a robust repellent effect on *T. absoluta* (*χ*^2^ = 8.1, *p* = 0.0044 and *χ*^2^ = 25.6, *p* < 0.0001, respectively). However, the predator *N. tenuis* displayed a contrasting response when exposed to these oils (Figure 1C,D). Indeed, *A. millefolium* and *A. sativum* oils were found to have a significant attractive activity to *N. tenuis* (*χ*^2^ = 4.9, *p* = 0.0268 and *χ*^2^ = 6.4, *p* = 0.0114, respectively). No preference was observed between the control and *R. officinallis*, *T. minuta* and *T. zygis* oils for *T. absoluta* and its predator, except the significant attractive behavior of *T. absoluta* on plants sprayed with *R. officinallis* (*χ*^2^ = 6.4, *p* = 0.0114) (Figure 1C).

### 2.2. Effect of PEOs on the T. absoluta-Infested Leaflets

**Figure 2:**
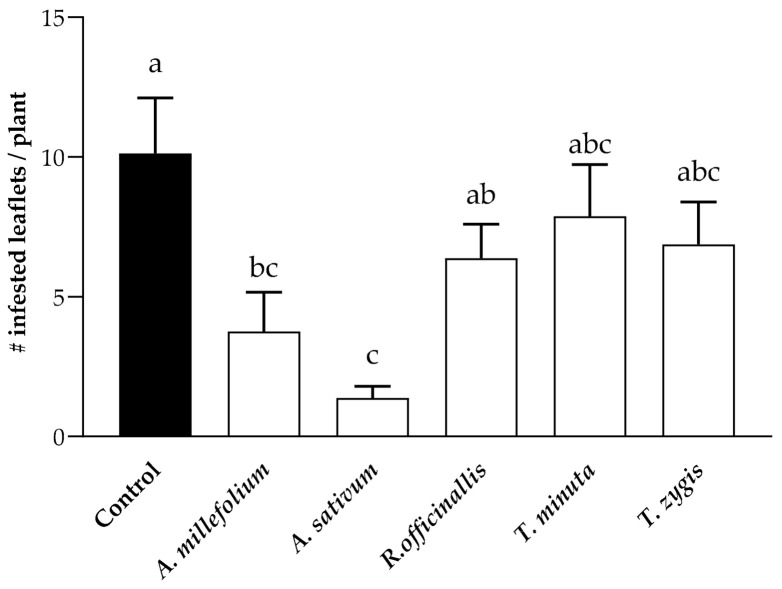
Number (mean ± SE) of *T. absoluta*-infested leaflets on tomato plants sprayed with different PEOs under greenhouse conditions. Bars marked with different lower-case letters are significantly different (Tukey’s test; *p* ≤ 0.05).

The number of leaflets infested by *T. absoluta* differed significantly on tomato plants sprayed with *A. millefolium*, *A. sativum*, *R. officinallis*, *T. minuta*, or *T. zygis*, and those left untreated (Figure 2). The number of *T. absoluta*-infested leaflets was significantly reduced in those plants sprayed with *A. millefolium* and *A. sativum* oils compared to the control treatment (*F*_5, 42_= 2.413, *p* = 0.0210).

### 2.3. VOCs Emitted by A. millefolium or A. sativum- Sprayed Tomato Plants

**Figure 3:**
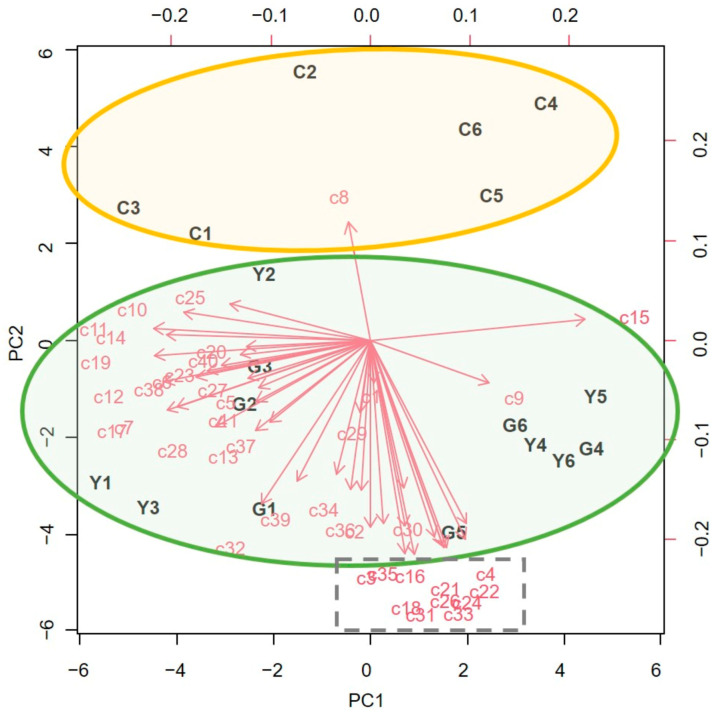
PCA of the proportion of the compounds (c1–c42, see Table 1 for respective compound names) detected in the volatile profiles of control (C), *Achillea millefolium* (Y), or *Allium sativum* (G) treated tomato plants. Samples of treated plants (Y and G) are grouped within the green ellipse, whereas the control samples are within the yellow ellipse. The compound characteristics of treated plants are grouped within the dotted grey box. Before the analysis, data (i.e., peak proportions) were transformed using the arcsin (sqrt x) function.

A total of 42 compounds were detected in the tomato plants sampled (Table 1). The volatile profiles were dominated by monoterpenes, regardless of the treatment applied to the plants, with β-phellandrene (c15), 2-carene (c10), and limonene (c14) being the major compounds (each accounting for more than 10% of the total chromatogram area). Notably, four compounds were found in the plants treated with PEOs but were not present in the control samples: (Z)-3-hexenyl propanoate (c21), (Z)-3-hexenyl butanoate (c26), (Z)-3-hexenyl-2-methylbutanoate (c31), and (Z)-3-hexenyl 2-methyl-(E)-2-butenoate (c33). The multivariate PCA revealed that the samples had different compositions between treated and control plants (Figure 3). The first two principal components, PC1 and PC2, correspond to the directions with the most significant amount of variation in the dataset, with eigenvalues of 1.29 and 0.99, respectively, accounting for 31.5% and 24.1% of the total data variability. The compounds responsible for the differences marked by PC1 are located on the right side of the plot (Figure 3) and include β-myrcene (c9) and β-phellandrene (c15). However, the mean proportion of these compounds was not significantly different among treatments, as shown in the ANOVA results (Table 1). The compounds responsible for the differences marked by PC2 are located at the bottom of the plot (Figure 3). This group includes the compounds that were only detected in the treated tomato plants (c21, c26, c31, and c33), regardless of the PEO used, as well as others that were detected in significantly lower proportions in the control plants: the green leaf alcohol Z-3-hexen-1-ol (c3), the aldehydes heptanal (c4), nonanal (c22), and decanal (c30), the monoterpene (Z)-β-ocimene (c16), and the alcohol 1-octanol (c18) (Table 1).

### 2.4. Defense Gene Expression in A. millefolium or A. sativum- Sprayed Tomato Plants

**Figure 4:**
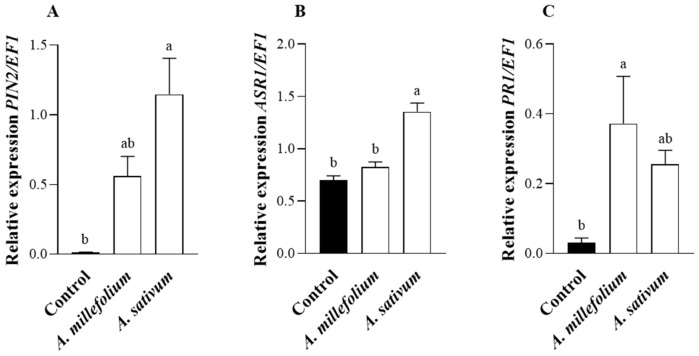
Transcriptional response of the defensive marker genes *PIN2* (**A**), *ASR1* (**B**), and *PR1* (**C**) of JA, ABA, SA, and signaling pathways, respectively, in tomato plants sprayed or not with *Achillea millefolium* or *Allium sativum* essential oils. The analysis was conducted 24 h after the spray. The means of four plants in each treatment ± SE are shown. Bars marked with different lower-case letters are significantly different (Tukey’s test; *p* ≤ 0.05).

To confirm the plant defense response to the PEO treatments, the transcriptional levels of the *PIN2*, *ASR1*, and *PR1* genes were studied in control and *A. millefolium* or *A. sativum* oil-treated tomato plants (Figure 4). The analysis revealed that the expression of *PIN2* and *ASR1* were significantly upregulated in tomato plants sprayed with *A. sativum* PEO. Meanwhile, the induction of *PR1* was significantly upregulated in tomato plants sprayed with *A. millefolium* PEO [*PIN2*, *ASR1,* and *PR1*: *F*_2, 14_ = 10.97, *p* = 0.002 (Figure 4A), *F*_2, 14_ = 31.39, *p* < 0.0001 (Figure 4B) and *F*_2, 14_ = 4.457, *p* = 0.0357 (Figure 4C), respectively].

### 2.5. Side-Effect of A. millefolium and A. sativum on N. tenuis

**Figure 5:**
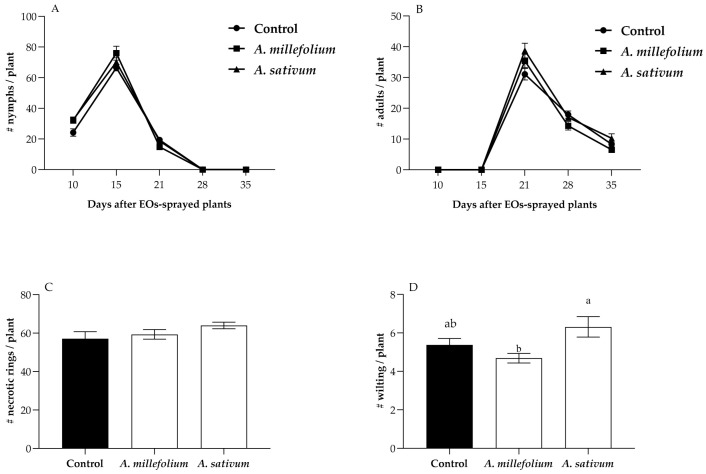
Performance of *Nesidiocoris tenuis* on plants previously treated with *Achillea millefolium* or *Allium sativum* essential oils. (**A**) Number (mean ± SE) of nymphs per plant; (**B**) Number (mean ± SE) of adults per plant; (**C**) Number (mean ± SE) of necrotic rings per plant; (**D**) Number (mean ± SE) of wilted plants. Tukey’s multiple range test determined the significance between treatments at *p* ≤ 0.05. Different lower-case letters are significantly different.

*Nesidiocoris tenuis* successfully established and reproduced when released on tomato plants previously sprayed with *A. millefolium* or *A. sativum* oils, compared to the control. No significant differences between the control and treated plants were found in the number of adults and nymphs of *N. tenuis* per tomato plant (*F*_2, 21_ = 5.201; *p* = 0.8424 and *F*_2, 21_= 2.192, *p* = 0.1822, respectively, Figure 5A,B). Furthermore, at the end of the cycle of this predator, neither the number of brown rings nor the number of wilting per plant (*F*_2, 23_ = 1.617, *p* = 0.2223; *F*_2, 23_ = 4.394, *p* = 0.1533, respectively) were significantly different between the sprayed plants and the control (Figure 5C,D).

## 3. Discussion

Plant essential oils (PEOs), extracted from various families such as Asteraceae, Amaryllidaceae, and Lamiaceae, are gaining popularity as a method for controlling insect pests. Our research found that PEOs from specific aromatic and medicinal plants can trigger a defense response in *S. lycopersicum* (cv. Moneymaker). The unique chemical composition of these PEOs was found to increase transcript levels of defense genes *PIN2*, *PR1*, and *ASR1*, which affects the preference and/or performance of *T. absoluta* and its predator, *N. tenuis*.

This study utilized a Y-tube olfactometer to demonstrate a high level of repellency in PEOs of *A. millefolium* and *A. sativum* (at a concentration of 0.05%) towards adult females of *T. absoluta*. Previous research has established that PEOs from specific plants can repel *T. absoluta*, such as the oils of two ethnobotanical *Ocimum* plants (*O. gratissimum* and *O. kilimandscharicum*), with *O. gratissimum* being more effective [28]. The behavioral responses of *T. absoluta* females also indicated clear repellency of watermelon odor (*Citrullus lanatus* L.) due to its GLV constituents, suggesting its potential use in an IPM “Push-Pull” system [29]. The oviposition repellence exhibited by *O. gratissimum* L. (Lamiaceae) and *O. basilicum* L. (Lamiaceae) PEOs is thought to be related to the masking effects of these oils on volatile tomato compounds, thereby preventing *T. absoluta* females from recognizing the presence of tomatoes [30]. More recently, a nanoformulation of *A. sativum* has shown promising results against *T. absoluta*, as the mean number of eggs laid per female was twice as low on leaves sprayed with this formulation compared to those on control leaves, leading to the conclusion that this oil is more effective against moth eggs than other PEOs [25].

PEOs may serve as a viable alternative to chemical insecticides, as they may be more compatible with natural enemies, which play a crucial role in reducing insect pest damage and reducing the need for large amounts of harmful insecticides [31]. In this study, the attractive olfactory response of the pest predator *N. tenuis* to *A. millefolium* and *A. sativum*-sprayed plants was significant compared to the unsprayed-plants. Still, understanding mirid predator orientation using natural bioactive compounds requires further studies to improve and develop pest control approaches for tomato pests [32].

The composition of PEOs was studied using GC-MS, and the major constituents were found to be the sesquiterpene β-caryophyllene (28.28%) for *A. millefolium* and diallyl trisulfide (39.93%) and diallyl disulfide (15.97%) for *A. sativum*. These molecules might target the receptor gamma-aminobutyric acid, which has been suggested to be the most sensitive target site in *T. absoluta*’s nervous system [33]. The abundance of caryophyllene and/or caryophyllene oxide in the PEOs of *Artemisia argyi* and *Salvia ballotiflora* has been found to be responsible for the insecticidal and repellent properties against the malaria vector *Anopheles sinensis* Wiedemann (Diptera: Culicidae) and the caterpillar of *Spodoptera frugiperda* Walker (Lepidoptera: Noctuidae) [34,35]. The natural optimization of the sesquiterpene biosynthetic pathway in wild-tomato germplasm to enhance the accumulation of 7-epi zingiberene has been shown to alter insect-choice behavior and improve defense in cultivated tomatoes [36]. The major constituents of the oil of *Ocimum gratissimum* (methyl eugenol (39.5%) and eugenol (29.7%)) were found to be significantly repellent towards *T. absoluta* adults. However, subtracting these compounds from a synthetic blend significantly decreased the repellency effect [28]. When searching for synergistic effects among constituents of PEOs, it is important to take into consideration the presence of other constituents, as previously reported when investigating the reasons behind the higher activity of Ajwain oil (*Carum copticum*, Apiaceae) against *T. absoluta* [37]. Results from this study and previous ones have shown slight quantitative and/or qualitative differences in the major components of *A. sativum* PEOs [25,38,39], with high toxicity observed against different orders of insects [38]. In fact, the PEO of *A. sativum* has been reported to inhibit acetylcholinesterase enzyme activity, acting individually or in synergy [40,41].

In this study, we provide new insights into the preference of *T. absoluta* for plant-PEO interactions by demonstrating that the repellence of *A. millefolium* and *A. sativum* PEOs is linked to activating the plant immune system through the JA, SA, and ABA pathways. Previous studies have shown that PEOs can, directly and indirectly, affect plant protection against insects. For example, candy mint (*Mentha* × *piperita* cv. Candy) and peppermint (*M.* × *piperita* L.) oils have been shown to increase the expression levels of the defense genes trypsin inhibitor and *PR1* in soybean leaves through histone modifications of their promoter regions [42]. JA and SA signaling pathways are key regulators of defense responses, with JA playing a particularly important role in plant defense against herbivorous arthropods [43]. The induction of resistance in tomatoes can vary greatly depending on the types of inducers [44], and these pathways may act individually, synergistically, or antagonistically depending on the herbivore [7]. Furthermore, some aromatic plants may also constitutively emit volatiles that can elicit defenses, such as the emission of terpenes in potato plants upon exposure to onion plant volatiles, which attract the herbivore enemy *Coccinella septempunctata* L. (Coleoptera: Coccinellidae) [45]. In our study, *A. sativum* and *A. millefolium* PEOs, or one or several of its constituents, also act as an elicitor to trigger the emission of HIPVs, specifically (Z)-3-hexen-1-ol, (Z)-3-hexenyl propanoate, (Z)-3-hexenyl butyrate, (Z)-β-ocimene, heptanal, nonanal and 1-octanol., which can be responsible for the behavioral responses observed in *T. absoluta* and *N. tenuis* behavior [32,46,47,48]. The C_6_ GLVs detected in our samples (alcohol and esters) are widely reported as messengers in tritrophic interactions by pointing out the presence of herbivores to their natural enemies [49]. Many terpenes, the largest class of volatiles produced by plants, have been reported to attract natural enemies and repel herbivores [50]. The monoterpene (Z)-β-ocimene is not an exception and was found to repel the herbivorous cereal beetle, *Oulema cyanella* Voet [51]. The aldehydes significantly promoted in our treated tomato plants are also known to attract and/or arrest beneficials, such as the parasitic wasp *Cotesia vestalis* by means of heptanal. Nonanal has also been reported as a component in HIPV volatile blends, attracting several species of natural enemies in open cotton fields [52]. The fatty alcohol 1-octanol was reported as having a strong repellent effect on fruit flies [53] and also as a potential oviposition deterrent in the Asian corn borer, *Ostrinia furnacalis* (*Guenée*) (Lepidoptera: Crambidae) [54].

In summary, this work demonstrates that PEOs can have a dual benefit in controlling arthropod pests. On one hand, there is the direct toxicity that PEOs can have on arthropod pests, and on the other, the effect of the defensive activation they produce in plants. This work shows that defensive activation induces the production of volatiles that repel and attract *T. absoluta* and *N. tenuis*, respectively. Furthermore, although we have not demonstrated it in this work, it is widely known that the activation of the JA and SA pathways can also trigger the production of compounds by plants that are toxic to arthropods [22]. Therefore, it would be interesting to discern this point in future studies. Based on our results, it is impossible to discriminate which of the two benefits has more weight, which will depend on each PEO, but it is evident that both are important.

Although this study yielded positive results, this work is a fundamental first step to addressing more practical work where the role of PEOs in the management of *T. absoluta* can be tested in field conditions. Based on the results of this work, the next step will be to select *A. millefolium* and *A. sativum* PEOs and to know their real potential for inclusion in current tomato pest management protocols. In this sense, it is also important to mention the good results obtained by Ricupero et al. [25] with garlic nanoencapsulations. The nanoencapsulation technology may improve the insecticidal properties of PEOs.

## 4. Materials and Methods

### 4.1. Plants, Plant Essential Oils, and Insects

*Solanum lycopersicum* (cv. Moneymaker) seeds were germinated in soil. After germination, the seedlings were transplanted into plastic pots (8 × 8 × 8 cm) and maintained without exposure to insecticides in a climatic chamber set at 25 °C, relative humidity of 65% ± 5%, and a photoperiod of 14:10 h (L:D) (approx. 2500 luxes).

Commercial essential oils of *A. millefolium*, *A. sativum*, *R. officinale*, *T. minuta*, and *T. zygis* were selected for this study (Supplementary Tables S1–S6). To prepare the formulated PEOs for their spraying, first, Tween 80 (Tween^®^ 80, Sigma-Aldrich, Markham, ON, Canada) was dissolved 2 *v*/*v* in bi-distilled water at room temperature. The mixture was shaken with a magnetic stirrer for 30 min to obtain a homogeneous solution. The PEOs were added gradually to the prepared Tween and mixed with a direct run stirrer for one hour to reach the following final concentrations: 0.05, 0.1, 0.5, 1, and 2.5%. After studying the possible phytotoxic effect of each of the five studied PEOs (Supplementary Materials Figure S1), it was decided to use concentrations of 0.05%.

*Tuta absoluta* individuals were obtained from colonies maintained at the Instituto Valenciano de Investigaciones Agrarias (IVIA) in Valencia, Spain. The colonies were reared on tomato plants in a growth chamber, kept in bugdorm cages (60 × 60 × 60 cm) (BugDorm-1 Insect Tents; MegaView Science Co., Ltd., Taichung, Taiwan), and maintained in an environmental chamber at a temperature of 25 ± 4 °C, relative humidity of 60% ± 15%, and a 14:10 h (L:D) photoperiod (approx. 2500 luxes).

*Nesidiocoris tenuis* were obtained from the mass rearing of Koppert Biological Systems, S.L. (Aguilas, Murcia, Spain). After receiving the insects, they were reared on bean pods (*Phaseolus vulgaris* L., Fabaceae) and kept for one day in a plastic cage (30 × 30 × 30 cm) (BugDorm-1 Insect Tents; MegaView Science Co., Ltd., Taichung, Taiwan) in a growth chamber at 25 ± 2 °C, relative humidity of 65% ± 10%, and a 14:10 h (L:D) photoperiod (approx. 2500 luxes). Predators were provided with frozen eggs of *Ephestia kuehniella* (Zeller) (Lepidoptera: Pyralidae) as supplementary food. The females and males of *N. tenuis* used in the greenhouse experiment came from cohorts of similar age and were prepared as previously described by Chinchilla-Ramirez et al. [55].

### 4.2. Olfactory Response of T. absoluta and N. tenuis to PEOs

After determining the phytotoxicity of the five essential oils on tomato plants, the olfactory preference of the herbivore pest *T. absoluta* and the predator *N. tenuis* was assessed using a Y-tube olfactometer (Analytical Research Systems, Gainesville, FL, USA). The olfactometer consisted of a 2.4-cm diameter Y-shaped glass tube with a 13.5-cm long base and two 5.75-cm long arms. The base of the Y-tube was connected to an air pump that produced a unidirectional airflow at 150 mL/min from the arms to the base of the tube. The arms were connected via plastic tubes to two identical glass jars (5-l volume), each containing a treated or a control plant. Each jar was connected to a flow meter and a water filter. Four 60-cm-long fluorescent tubes (OSRAM, L18 W/765, OSRAM GmbH, Munich, Germany) were positioned 40 cm above the arms. The light intensity over the Y-tube was measured with a ceptometer (LP-80 AccuPAR, Decagon Devices, Inc., Pullman, WA, USA) at 2516 lux. The environmental conditions in the Y-tube experiments were 23 ± 2 °C and 60% ± 10% relative humidity [56].

Two experiments were conducted to distinguish between each essential oil’s inherent attraction or repellence effect and the effect that each essential oil could have on the activation of plant responses. In the first experiment, the olfactory responses of *T. absoluta* and *N. tenuis* to the essential oils were tested by placing a piece of filter paper (3 cm in diameter) with 20 µL of the chosen essential oil in one arm of the Y-tube olfactometer. In the other arm, a filter paper with 20 µL of 2% Tween was introduced as a control. In the second experiment, the olfactory responses of both female insects were tested by placing a tomato plant that was previously sprayed with one of the essential oils in one arm of a Y-tube olfactometer, while in the other arm, a control plant treated only with Tween was introduced. The sprayed plants were kept for 24 h in isolated climatic chambers to avoid interference and were maintained at 25 ± 2 °C, 65% ± 10% relative humidity, and a 14:10 h (L:D) photoperiod. Each plant was used in just 10 repetitions. *Tuta absoluta* and *N. tenuis* were released into the base arm of the Y-tube olfactometer individually using a small aspirator. The Y-tube olfactometer was inverted to avoid direction errors after releasing 5 tested females. Each tested female’s response was considered when it reached the end of one arm. Females that did not choose either side of the two arms, after 15 min were considered non-responders and were excluded from statistical analysis. Forty responses were conducted for each of the tested combinations.

### 4.3. The Suitability of PEOs in Reducing T. absoluta-Infested Leaflets

The experiment was carried out at IVIA, under greenhouse conditions, with a temperature of 25 °C ± 1 °C, 65% ± 10% relative humidity, and a natural photoperiod (approximately 14:10 h, L:D). Tomato plants were sprayed with each of the PEOs as described before. Each plant was enclosed in a plastic cage (24.5 × 24.5 × 630 cm) (BugDorm-1 Insect Tents; MegaView Science Co., Ltd., Taichung, Taiwan), and two pairs (male and female) of *T. absoluta* adults were released per plant. Tween-sprayed plants served as a control. Tomato plants were sprayed only once before the release of the insects. Eight replicates (one plant per replicate) were considered for each treatment. The plants were distributed in the greenhouse following a randomized block experimental design with 4 blocks (each block contained two replicates of each treatment). After 14 days, the number of infested leaflets (leaves with damage from galleries produced by larvae of *T. absoluta*.) induced by *T. absoluta* was recorded.

### 4.4. Headspace Collection and Analysis of Volatile Compounds Induced by Plants Exposed to PEOs

Volatile compounds (VOCs) were collected from both control *S. lycopersicum* plants and *S. lycopersicum* plants exposed to 0.05% *A. millefolium* and *A. sativum* oils using HS-SPME in static conditions. Individual tomato plants were placed in 5-L glass jars (25 cm high by 17.5 cm diameter) with a 10 cm open mouth and a ground glass flange to fit the cover with a clamp. The cover had a 29/32 neck on top to fit a glass adapter with a GL14 screw cap with a 12-mm red polytetrafluoroethylene (PTFE)/silicone septum. A sample of the headspace in the jar was taken using an SPME holder equipped with a polydimethylsiloxane/divinylbenzene fiber (PDMS/DVB, 65 μm film thickness; (Supelco Inc., Torrance, CA, USA). The SPME fibers were conditioned in a GC injection port set at 250 °C for 10 min before volatile sampling. For the sampling, the SPME needle was inserted through the septum mentioned above, and the fiber was exposed to each sample headspace for 1 h. After this period, the fibers were removed and inserted into the GC injection port to desorb the volatiles for chromatographic analysis.

Gas chromatography coupled with mass spectrometry (GC-MS) using a Clarus 690 GC and Clarus SQ 8T MS detector (PerkinElmer Inc., Waltham, MA, USA) analyzed the volatiles collected with the SPME fibers. The SPME fibers were desorbed for 2 min into the GC injection port set in splitless mode at 250 °C. The column used was a ZB-5MS (30 m × 0.25 mm i.d., 0.25 μm film thickness) fused silica capillary column (Phenomenex Inc., Torrance, CA). The oven was held at 40 °C for 4 min and then programmed to increase 8 °C/min to reach 250 °C and held for 2 min. Helium was used as the carrier gas with a flow rate of 1 mL/min. The detection was performed in the EI mode (70 eV) with the ionization source set at 200 °C. The spectrum acquisition was completed in full scan mode (mass range m/z 33−450), and chromatograms and spectra were recorded using GC-MS Turbomass software v. 6.1.2 (PerkinElmer Inc.). Compounds were identified by comparing their mass spectra with those of pure standards when available and, tentatively, based on high probability matches (>80%) according to the NIST MS Search routine (NIST Mass Spectral Search Program for the NIST\EPA\NIH Mass Spectral Library, version 2.4, build 3/2020).

### 4.5. Plant Gene Expression

To evaluate the effect of spraying with *A. millefolium* and *A. sativum* PEOs on the activation of the plant immune system, the transcriptional response of the proteinase inhibitor II (*PIN2*), the pathogenesis-related protein precursor 1 (*PR1*), and the abscisic acid stress ripening 1 (*ASR1*), defensive genes related to JA, SA, and ABA signaling pathways, respectively, were studied. Under greenhouse conditions (the same as described above), tomato plants were separately maintained in three 60 × 60 × 60 cm plastic cages (BugDorm-2), each containing five plants (cv. Moneymaker), sprayed either with 0.05% *A. millefolium* or 0.05% *A. sativum* essential oils or Tween-treated control plants.

Twenty-four hours after the spray, the apical part of each plant was collected and grounded in liquid nitrogen. Total RNA was isolated using NZYol (NZYTech, Lisboa, Portugal) based extraction. Five μg of each RNA sample was treated with TURBO DNA-freeTM Kit (AmbionR, Life Technologies, Carlsbad, CA, USA) in a 25 μL reaction to eliminate DNA contamination. Reverse transcription was achieved, and cDNA was synthesized using a Prime ScriptTM RT Reagent Kit (TAKARA Bio, San Jose, CA, USA). Purified cDNA samples were diluted to 500 ng with RNase-free water, and real-time PCR amplification was performed in the LightCycler^®^ 480 System (Roche Molecular Systems, Inc., Basel, Switzerland), using NZYSupreme qPCR Green Master Mix (2x) (NZYTech, Lisboa, Portugal). The qRT-PCR reaction mixture of 10 μL containing 2 μL of template cDNA and 0.5 μM of each primer was incubated for 5 min at 95 °C, followed by 40 cycles of 15 s at 95 °C, 30 s at 58 °C and 30 s at 72 °C and followed by melting curve analysis. Forward and reverse nucleotides sequence of the defensive genes *PIN2*, *PR1* and *ASR1* were 5′-GAAAATCGTTAATTTATCCCAC-3′/5′-ACATACAAACTTTCCATCTTTA-3′; 5′-CTCATATGAGACGTCGAGAAG-3′/5′-GGAAACAAGAAGATGCAGTACTTAA-3′ and 5′-ACACCACCACCACCACCTGT-3′/5′-GTGTTTGTGTGCATGTTGTGGA-3′, respectively. Elongation factor 1 (*EF1)* was used as a housekeeping gene (5′-GATTGGTGGTATTGGAACTGTC-3′/5′-AGCTTCGTGGTGCATCTC-3′).

### 4.6. Side-Effects of A. millefolium and A. sativum on N. tenuis

Given the effectiveness of *A. millefolium* and *A. sativum* in controlling *T. absoluta*, their potential impact on *N. tenuis* was evaluated under greenhouse conditions. The greenhouse conditions were 25 ± 2 °C, 65% ± 10% RH, and a natural photoperiod (approximately 14 L:10 D). The experiment consisted of 12 cages (75 cm × 75 cm × 115 cm) (BugDorm insect tents, MegaView Science Co., Ltd., Taichung, Taiwan), with four replicates per treatment (*A. millefolium*, *A. sativum* treatments, and a control (un-sprayed plants). Each cage represented one replicate and contained eight tomato plants. A randomized block design with four blocks was followed.

Tomato plants were sprayed with *A. millefolium* or *A. sativum,* using a handle sprayer until runoff (approximately 25 mL of each oil solution per plant). Two couples (male and female) of *N. tenuis* were released per plant 24 h after the treatments. During the experiment, eggs of *E. kuehniella* were provided as a food source for *N. tenuis* twice a week. Following ten days, the predators were removed using a manual aspirator, and the number of nymphs and newly emerged adults were counted throughout the experiment. At the end of the experiment, the number of necrotic rings was also recorded [57].

### 4.7. Statistical Analysis

Chi-square (*χ*^2^) goodness of fit tests based on a null model was used to analyze data collected from the olfactory responses, where the odor sources were selected with equal frequency. Individuals that did not make a choice were excluded from the statistical analysis. Data obtained from the HIPVs quantification, gene expression, and greenhouse on *N. tenuis* damage were analyzed using one-way ANOVA, with Tukey’s posthoc test at *p* < 0.05. Data from the population dynamics of *N. tenuis* in the greenhouse were analyzed using repeated measures ANOVA with a significance level of *p* < 0.05. All statistical analyses were conducted using GraphPad Prism 9 for Windows (GraphPad Software, San Diego, CA, USA).

Principal component analysis (PCA) was applied to visualize the differences in the proportion of each compound in three experimental conditions (control, *A. millefolium*, and *A. sativum*) through score and loading plots. The chromatographic peak areas of all detected compounds were integrated for each sample, and the proportion of each compound was calculated relative to the total chromatogram area. The data were arranged in a matrix of 18 rows (samples) and 42 columns (chemical compounds as variables). To normalize the data, area values were transformed using the angular transformation (arcsin(sqrt(x))). PCA was performed using the *prcomp* function, and the number of principal components was determined by evaluating their eigenvalues (λ) and proportion of variances with the *get_eigenvalue* function in the factoextra package. The scores were visualized using the *ggplot* function in the ggplot2 package. All data analysis was conducted using R version 4.2.0 [58]. Furthermore, one-way ANOVA was used to evaluate the significance of differences observed between treatments (LSD posthoc test at *p* < 0.05) using Statgraphics Centurion 18 v. 18.1.13 (Statgraphics Technologies Inc., The Plains, VA, USA).

## 5. Conclusions

Our research indicates that utilizing plant extracts as pesticides could be a viable alternative to synthetic options. Plant extracts possess several advantages over synthetic pesticides, such as being more environmentally friendly, less toxic to non-target organisms, and more cost-effective. However, there are still obstacles that must be overcome for the widespread adoption of plant extracts as pesticides. These include issues with consistency in the quality and composition of plant extracts, a need for a deeper understanding of the mode of action of many plant extracts, and a need for standardization. Despite these challenges, research in this field is ongoing, and further studies are necessary to understand and address these obstacles fully. The future of using plant extracts as pesticides is optimistic and has the potential to improve the sustainability of agricultural systems significantly.

## Figures and Tables

**Table 1:** Volatile compounds detected in samples of tomato plants treated with essential oils of *Achillea millefolium* and *Allium sativum* relative to control tomato plants (mean percentages ^1^ of 6 replicates).

rt (min) ^2^	Code ^3^	Name	Treatment			ANOVA
			Control	*A. millefolium*	*A. sativum*	
4.77	c1	Z-3-hexenal	0.079 ± 0.053 a	0.056 ± 0.015 a	0.033 ± 0.012 a	F _2,14_ = 0.44, *p* = 0.6528
4.83	c2	hexanal	0.064 ± 0.014 a	0.156 ± 0.028 a	0.080 ± 0.015 a	F _2,15_ = 0.66, *p* = 0.5333
6.72	c3	Z-3-hexen-1-ol	0.087 ± 0.066 a	0.336 ± 0.066 b	0.179 ± 0.054 ab	F _2,14_ = 5.59, *p* = 0.0165
8.14	c4	heptanal	0.003 ± 0.001 a	0.089 ± 0.034 b	0.089 ± 0.025 b	F _2,13_ = 4.61, *p* = 0.0306
8.76	c5	3-thujene	0.047 ± 0.007 a	0.062 ± 0.009 a	0.051 ± 0.006 a	F _2,15_ = 0.99, *p* = 0.3929
8.96	c6	α-pinene	2.529 ± 0.286 a	2.838 ± 0.346 a	3.083 ± 0.385 a	F _2,15_ = 0.61, *p* = 0.5542
9.94	c7	3,7,7-trimethyl-1,3,5-cycloheptatriene	3.024 ± 0.663 a	3.712 ± 0.693 a	3.185 ± 0.436 a	F _2,15_ = 0.34, *p* = 0.7139
10.10	c8	β-pinene	0.427 ± 0.203 a	0.339 ± 0.149 a	0.171 ± 0.022 a	F _2,15_ = 0.97, *p* = 0.4009
10.40	c9	β-myrcene	2.170 ± 0.253 a	1.929 ± 0.276 a	2.586 ± 0.393 a	F _2,15_ = 1.05, *p* = 0.3741
10.64	c10	2-carene	24.257 ± 3.267 a	23.441 ± 2.950 a	24.054 ± 2.653 a	F _2,15_ = 0.02, *p* = 0.9796
10.80	c11	α-phellandrene	6.610 ± 0.981 a	6.182 ± 1.080 a	5.917 ± 0.540 a	F _2,15_ = 0.12, *p* = 0.8876
11.05	c12	α-terpinene	2.361 ± 0.521 a	2.340 ± 0.532 a	2.426 ± 0.366 a	F _2,15_ = 0.03, *p* = 0.9700
11.23	c13	*p*-cymene	0.262 ± 0.057 a	0.446 ± 0.079 a	0.465 ± 0.092 a	F _2,15_ = 2.65, *p* = 0.1034
11.42	c14	limonene	16.916 ± 3.394 a	18.124 ± 5.063 a	17.761 ± 5.033 a	F _2,15_ = 0.00, *p* = 0.9979
11.49	c15	β-phellandrene	36.615 ± 8.794 a	32.838 ± 11.003 a	33.174 ± 8.396 a	F _2,15_ = 0.08, *p* = 0.9248
11.70	c16	(Z)-β-ocimene	0.714 ± 0.157 a	1.128 ± 0.104 b	1.276 ± 0.141 b	F _2,15_ = 4.16, *p* = 0.0364
11.98	c17	γ-terpinene	0.408 ± 0.070 a	0.454 ± 0.087 a	0.455 ± 0.040 a	F _2,15_ = 0.20, *p* = 0.8207
12.24	c18	1-octanol	0.000 ± 0.000 a	0.033 ± 0.005 b	0.027 ± 0.005 b	F _2,11_ = 19.24, *p* = 0.0003
12.56	c19	terpinolene	0.657 ± 0.130 a	0.652 ± 0.170 a	0.609 ± 0.098 a	F _2,15_ = 0.02, *p* = 0.9765
12.66	c20	3,4-dimethylstyrene	0.033 ± 0.009 a	0.039 ± 0.005 a	0.029 ± 0.006 a	F _2,15_ = 0.61, *p* = 0.5559
12.81	c21	(Z)-3-hexenyl propanoate	nd	0.011 ± 0.003 a	0.027 ± 0.010 a	F _1,10_ = 2.32, *p* = 0.1590
12.94	c22	nonanal	0.055 ± 0.007 a	1.369 ± 0.385 b	1.119 ± 0.276 b	F _2,15_ = 11.75, *p* = 0.0009
13.20	c23	isoterpinolene	0.174 ± 0.040 a	0.186 ± 0.052 a	0.126 ± 0.035 a	F _2,15_ = 0.66, *p* = 0.5309
14.06	c24	2-nonenal	0.008 ± 0.003 a	0.036 ± 0.009 a	0.026 ± 0.006 a	F _2,12_ = 2.93, *p* = 0.0922
14.42	c25	unknown	0.036 ± 0.010 a	0.023 ± 0.006 a	0.034 ± 0.005 a	F _2,15_ = 0.97, *p* = 0.4004
14.54	c26	(Z)-3-hexenyl butanoate	nd	0.039 ± 0.013 a	0.069 ± 0.024 a	F _1,10_ = 0.82, *p* = 0.3862
14.61	c27	dill ether	0.093 ± 0.008 a	0.088 ± 0.018 a	0.102 ± 0.021 a	F _2,15_ = 0.13, *p* = 0.8765
14.71	c28	methyl salicylate	0.021 ± 0.007 a	0.053 ± 0.020 a	0.033 ± 0.016 a	F _2,15_ = 1.09, *p* = 0.3602
14.81	c29	dodecane	0.032 ± 0.004 a	0.040 ± 0.004 a	0.031 ± 0.004 a	F _2,15_ = 1.59, *p* = 0.2370
14.93	c30	decanal	0.037 ± 0.008 a	0.099 ± 0.012 b	0.062 ± 0.010 c	F _2,15_ = 11.01, *p* = 0.0011
15.38	c31	(Z)-3-hexenyl-2-methylbutanoate	nd	0.009 ± 0.001 a	0.015 ± 0.005 a	F _1,10_ = 0.60, *p* = 0.4571
16.76	c32	isoascaridol	0.043 ± 0.005 a	0.054 ± 0.007 ab	0.065 ± 0.004 b	F _2,15_ = 4.14, *p* = 0.0369
17.01	c33	(Z)-3-hexenyl 2-methyl-(E)-2-butenoate	nd	0.011 ± 0.003 a	0.017 ± 0.002 a	F _1,10_ = 0.11, *p* = 0.7487
17.27	c34	δ-elemene	0.175 ± 0.039 a	0.214 ± 0.059 a	0.387 ± 0.062 b	F _2,15_ = 4.04, *p* = 0.0395
17.43	c35	epoxide	0.011 ± 0.001 a	0.018 ± 0.002 b	0.029 ± 0.003 c	F _2,15_ = 18.18, *p* = 0.0001
18.19	c36	β-elemene	0.019 ± 0.003 a	0.026 ± 0.007 ab	0.041 ± 0.005 b	F _2,15_ = 3.94, *p* = 0.0423
18.30	c37	tetradecane	0.018 ± 0.005 a	0.027 ± 0.006 a	0.029 ± 0.009 a	F _2,15_ = 0.79, *p* = 0.4727
18.73	c38	β-caryophyllene	1.885 ± 0.283 a	2.310 ± 0.559 a	1.985 ± 0.262 a	F _2,15_ = 0.13, *p* = 0.8754
19.04	c39	sesquiterpene 1	0.054 ± 0.005 a	0.071 ± 0.010 a	0.064 ± 0.006 a	F _2,15_ = 1.14, *p* = 0.3455
19.61	c40	sesquiterpene 2	0.057 ± 0.012 a	0.055 ± 0.014 a	0.033 ± 0.006 a	F _2,15_ = 1.25, *p* = 0.3138
19.71	c41	sesquiterpene 3	0.041 ± 0.007 a	0.038 ± 0.009 a	0.040 ± 0.007 a	F _2,15_ = 0.07, *p* = 0.9323
20.97	c42	4,8,12-trimethyltrideca-1,3,7,11-tetraene	0.016 ± 0.005 a	0.038 ± 0.026 a	0.026 ± 0.009 a	F _2,10_ = 0.11, *p* = 0.8949

^1^ Percentage (mean ± SE) of each compound according to the total chromatogram area; nd = not detected; for each compound, means followed by the same letter are not significantly different (LSD test, *p* > 0.05); ^2^. Retention time (min); ^3^ Compound code according to PCA.

## Data Availability

All data included in the main text.

## Supplementary Materials

The following supporting information can be downloaded at: https://www.mdpi.com/article/10.3390/plants12050985/s1, Supplementary Materials, Figure S1: The phytotoxicity index (mean ± SE) of the essential oils of *Achillea millefolium* (A), *Allium sativum* (B), *Rosmarinus officinalis* (C), *Tagetes minuta* (D), and *Thymus zygis* (E) on tomato growth after 14 days of treatment and compared to the control (Tween 2%).; Table S1: Plant essential oils tested in this study; Table S2: Essential oil composition of *Achillea millefolium* identified by GC-MS; Table S3: Essential oil composition of *Allium sativum* identified by GC-MS, Table S4: Essential oil composition of *Rosmarinus officinallis* identified by GC-MS, Table S5: Essential oil composition of *Rosmarinus officinallis* identified by GC-MS, Table S6: Essential oil composition of *Thymus zygis* identified by GC-MS.
