# Supplementary material for: The Dual Benefit of Plant Essential Oils against Tuta absoluta
Source: Plants (Basel). 2023 Feb 21;12(5):985. doi: 10.3390/plants12050985 (PMC10005231; doi:10.3390/plants12050985)
Supplement: Supplementary file 1 [file plants-12-00985-s001.zip › plants-2219052-supplementary.pdf]

The dual benefit of plant essential oils against *Tuta absoluta*

Saoussen Ben Abdallah[1,2], Chaymaa Riahi[2], Sandra Vacas[3], Vicente Navarro-Llopis[3], Alberto Urbaneja[2] and Meritxell Pérez-Hedo[2#]

[1]*Southwest Florida Research and Education Center. 2685 FL-29, Immokalee, FL 34142, (USA)*

[2]*Instituto Valenciano de Investigaciones Agrarias (IVIA), Centro de Proteccion Vegetal y Biotecnologia, CV-315, Km 10.7, 46113 Moncada, Valencia, Spain*

[3]*Centro de Ecología Química Agrícola – Instituto Agroforestal del Mediterráneo. Universitat Politècnica de València. Camino de Vera s/n, 46022 Valencia, Spain*

Supplementary materials, tables & figures

Plant essential oils

The following PEOs were used [yarrow (*Achillea millefolium*, Asteraceae), garlic (*Allium sativum*, Amaryllidaceae), rosemary (*Rosmarinus officinalis*, Lamiaceae), marigold (*Tagetes minuta*, Asteraceae) and thyme (*Thymus zygis*, Lamiaceae)] (**Supp. Table S1**).

Analysis of plant essential oil by gas chromatography–mass spectrometry

GC-MS conditions. The analyses of the PEO samples were performed by using a Clarus 690 GC coupled to a Clarus SQ 8T mass spectrometer (Perkin Elmer, Inc., Wellesley, PA, USA), equipped with a ZB-5MS fused-silica capillary column (30 m x 0.25 mm i.d. x 0.25 µm film thickness; Phenomenex Inc., Torrance, CA, USA). The injector was set at 250 ºC and the oven was held at 60 °C for 5 min, raised at 3 ºC/min up to 180 ºC and then programmed at 20 °C/min to reach 280 °C, held for 10 min. Helium was used as the carrier gas with a flow of 1 ml/min. The detection was performed in the EI mode (70 eV) with the ionization source set at 200 °C. The spectrum acquisition was performed in full scan mode (mass range m/z 33−450) and chromatograms and spectra were recorded by means of GC-MS Turbomass software v. 6.1.2 (PerkinElmer Inc.). Compounds were identified by comparing their mass spectra with those of pure standards when available and, tentatively, based on high probability matches (> 80 %) according to the NIST MS Search routine (NIST Mass Spectral Search Program for the

NIST\EPA\NIH mass Spectral Library, version 2.4, build 3/2020). For each PEO, the chromatographic peak areas of all detected compounds were integrated and the proportion of each compound regarding the total chromatogram area was calculated.

Essential oils composition. The detailed chemical composition of the tested PEOs are shown in Tables S2-S6. The composition of *A. millefolium* consisted of 70 constituents, with sesquiterpene compounds making up 57 % and monoterpenoids 39 % as the major compounds (**Supp. Table S2**). *Allium sativum* was made up of 38 constituents, primarily sulfur compounds, including diallyl trisulfide (39.93 %), diallyl disulfide (15.97 %), allyl methyl trisulfide (14.06 %), and diallyl tetrasulfide (7.41 %) as the major compounds (**Supp. Table S3**). *Rosmarinus officinalis* was composed of 51 constituents, mostly monoterpenoids (91 %) as the major compounds (**Supp. Table S4**). *Tagetes minuta* was made up of 54 constituents, with the monoterpenes Z-β-ocimene and dihydrotagetone accounting for 63 % of the total chromatogram area (**Supp. Table S5**). *Thymus zygis* was composed of 56 constituents, primarily monoterpenoids (94 %) as the major compounds (**Supp. Table S6**).

The phytotoxic effect of PEOs on tomato plants

The phytotoxic effect of the prepared concentrations (0.05%, 0.1%, 0.5%, 1%, and 2.5%) of each polyethylene oxide (PEO) was evaluated on tomato plants for two weeks, using the methodology described by **Campolo et al. (2017)**. The plants were sprayed with the five PEOs using a handheld sprayer until runoff (approximately 25 ml of each oil solution per plant). Plants treated only with 2% Tween served as control. The tomato plants were kept in a greenhouse at a temperature of 22 ± 3°C, relative humidity of 55 + 5%, and a photoperiod of 14:10 hours approximately (L:D). The plants were fertilized once a week with a mineral fertilizer during the experiment. The effect of the PEOs on the phenotype of the tomato plants was evaluated every 3 days for two weeks by recording the number of damaged leaflets and the severity of the damage. The severity was categorized as follows: zero (no foliar burn and/or wilting with maintenance of green color), one (partially damaged leaf with up to 25% burn

and/or wilting), two (leaves with 25 to 50% burn and/or wilting), three (more than 50% dead leaves), and four (totally dead leaves). The phytotoxic index (Pi) was calculated using the following formula.

$$P_i = \sum_{j=0}^{n} \left(\frac{\mathrm{DLJ}}{\mathrm{TL}} * \frac{DC}{n-1}\right)$$

Where *j* is the damaged severity class, *DL* is the number of damaged leaves for each damage severity class *j*, *TL* is the total number of sprayed leaves.

The results revealed that the phytotoxic index (*Pi*) increased significantly in a dose-dependent manner when tomato plants were sprayed with 0.1%, 0.5%, 1%, and 2.5% of all the tested oils (Supp. Figure 1). In contrast, tomato plants sprayed with 0.05% of each oil showed little to no phytotoxic effect.

Table S1. Plant essential oils tested in this study**.**

Essential oil trade name	Family	Scientific name	Plant extracted from	Extraction method	Manufacturer
**Yarrow**	Asteraceae	*Achillea Millefolium*	Aerial part	Steam distillation	Eco Aurous (London, UK)
**Garlic**	Amaryllidaceae	*Allium sativum*	Bulb	Distillation	Paranarom (Miami, FL)
**Rosemary**	Lamiaceae	*Rosmarinus officinalis*	Aerial part	Distillation	Essenciales (Barcelone, ES)
**Marigold**	Asteraceae	*Tagetes minuta*	Flower	Distillation	deve herbes (New delhi, IN)
**Thyme**	Lamiaceae	*Thymus zygis*	Aerial part		Aroma-Labs (Lyon, FR)

Table S2. Essential oil composition of *Achillea millefolium* identified by GC-MS.

rt (min)[1]	compound	% TIC[2]
6.85	tricyclene	0.27
6.98	2-thujene	0.04
**7.27**	**α-pinene**	**2.68**
**7.91**	**camphene**	**1.33**
**8.85**	**sabinene**	**1.26**
**9.06**	**β-pinene**	**4.68**
9.58	β-myrcene	0.05
10.30	α-phellandrene	0.01
10.40	3-carene	0.01
11.14	p-cymene	0.78
**11.35**	**limonene**	**1.01**
**11.49**	**eucalyptol**	**5.32**
12.18	trans-β-ocimene	0.01
12.70	γ-terpinene	0.42
13.96	terpinolene	0.34
14.10	fenchone	0.01
14.91	α-campholenal	0.32
15.33	1.5.5-Trimethylbicyclo[2.2.1]heptan-2-ol	0.04
**16.90**	**camphor**	**16.11**
**17.64**	**exo-borneol**	**1.98**
18.04	endo-borneol	0.18
19.15	α-terpineol	0.95
19.37	γ-terpineol	0.11
23.31	bornyl acetate	0.50
25.96	α-cubebene	0.21
26.14	eugenol	0.38
**26.55**	**neryl acetate**	**1.32**
27.16	α-copaene	0.64
28.36	Z-β-caryophyllene	0.05
**29.05**	**β-caryophyllene**	**28.28**
29.33	sesquiterpene	0.10
29.96	vestitenone	0.11
30.22	α-himachalene	0.13
**30.46**	**α-caryophyllene**	**3.92**
31.39	(4aS.9aR)-3.5.5.9-Tetramethyl-2.4a.5.6.7.9a-hexahydro-1H-benzo[7]annulene	0.09
32.09	α-selinene	0.05
32.26	β-himachalene	0.58
32.57	E.E-alpha-farnesene	0.16
33.01	β-cadinene	0.22
33.13	calamene	0.12
33.23	γ-dehydro-ar-himachalene	0.06
33.95	trans-α-bisabolene	0.05
34.20	sesquiterpene epoxide 1	0.10
35.18	caryophyllen alcohol	0.07
**35.42**	**epoxycaryophyllene**	**1.38**
36.17	longiborneol	0.13
36.47	humulene 6.7-epoxide	0.12

36.82	sesquiterpene epoxide 2	0.18
37.20	himachalol	0.10
37.35	unknown	0.15
37.50	10.10-dimethyl-2.6-dimethylenebicyclo[7.2.0]undecan-5-ol	0.16
37.61	unknown	0.14
37.82	unknown	0.28
38.11	sesquiterpene alcohol 1	0.23
38.20	sesquiterpene alcohol 2	0.17
38.26	unknown	0.22
38.58	allohimachalol	0.46
38.69	atlantone isomer	0.42
38.75	unknown	0.62
**39.40**	**Z-γ-atlantone**	**4.55**
**39.72**	**deodarone**	**1.17**
**39.83**	**E-γ-atlantone**	**4.81**
**40.38**	**Z-α-atlantone**	**1.43**
41.04	sesquiterpene alcohol 3	0.22
41.51	α-bisabolol oxide A	0.16
41.92	sesquiterpene alcohol 4	0.10
**42.48**	**E-α-atlantone**	**7.30**
42.61	unknown	0.22
44.59	6-dehydropetasol	0.14
45.04	unknown	0.10

[1] retention time (rt)

[2] Percentage of each compound according to the total chromatogram area.

Table S3. Essential oil composition of *Allium sativum* identified by GC-MS.

rt (min)[1]	compound	% TIC[2]
**4.81**	**diallyl sulfide**	**3.10**
5.63	(Z)-allyl(prop-1-en-1-yl)sulfane	0.06
5.72	(E)-allyl(prop-1-en-1-yl)sulfane	0.02
6.16	3,4-dimethylthiophene	0.05
**6.61**	**allyl methyl disulfide**	**2.92**
7.10	methyl cis-1-propenyl disulfide	0.26
7.43	methyl trans-1-propenyl disulfide	0.47
8.09	3H-1,2-dithiole	0.03
8.66	dimethyl trisulfide	0.97
12.07	ethyl 2-(methylthio)acetate	0.02
13.04	1-(butylsulfanyl)-1-propene	0.03
**13.75**	**diallyl disulfide**	**15.97**
**14.35**	**Z-1-propenyl 2-propenyl disulfide**	**3.01**
**14.67**	**E-1-propenyl 2-propenyl disulfide**	**5.44**
**16.45**	**allyl methyl trisulfide**	**14.06**
17.15	4-methyl-1,2,3-trithiolane	0.07
17.39	methyl cis-1-propenyl trisulfide	0.06
17.65	methyl trans-1-propenyl trisulfide	0.10
18.74	3-vinyl-1,2-dithi-4-ene	0.13
19.96	3-vinyl-1,2-dithiacyclohex-5-ene	0.72
23.26	sec-butyl-(Z)-propenyl-disulfide	0.25
23.58	(methylthio)dimethyl sulfoxide	0.17
**24.08**	**diallyl trisulfide**	**39.93**
24.50	2-propenyl propyl trisulfide	0.20
24.82	allyl (Z)-prop-1-enyl trisulfide	0.14
25.06	allyl (E)-prop-1-enyl trisulfide	0.28
26.75	5-methyl-1,2,3,4-tetrathiane	0.13
**27.32**	**sulphur compound**	**2.42**
31.01	unknown	0.09
31.20	unknown	0.07
33.26	2,4,5,7-tetrathiaoctane	0.06
**33.90**	**diallyl tetrasulfide**	**7.41**
34.27	1-(1-(prop-1-en-1-ylthio)propyl)-2-propyldisulfane	0.11
41.90	sulphur compound	0.29
43.75	1-allyl-3-(2-(allylthio)propyl)trisulfane	0.21
44.36	sulphur compound	0.35
46.11	sulphur compound	0.26
47.10	sulphur compound	0.17

[1] retention time (rt)

[2] Percentage of each compound according to the total chromatogram area.

Table S4. Essential oil composition of *Rosmarinus officinallis* identified by GC-MS.

rt (min)[1]	compound	% TIC[2]
6.75	tricyclene	0.17
6.88	2-thujene	0.32
**7.18**	**α-pinene**	**10.92**
**7.83**	**camphene**	**4.38**
7.97	dehydrosabinene	0.04
8.77	sabinene	0.12
**8.98**	**β-pinene**	**7.31**
9.08	1-octen-3-ol	0.13
9.31	3-octanone	0.13
**9.50**	**β-myrcene**	**1.32**
9.83	3-octanol	0.01
10.11	pseudolimonene	0.02
10.22	α-phellandrene	0.12
10.32	3-carene	0.19
10.71	α-terpinene	0.24
**11.07**	**p-cymene**	**2.54**
**11.29**	**limonene**	**3.50**
**11.49**	**eucalyptol**	**42.79**
11.63	Z-β-ocimene	0.03
12.11	E-β-ocimene	0.04
12.63	γ-terpinene	0.41
13.19	sabinene hydrate	0.07
13.89	terpinolene	0.24
14.64	linalool	0.65
15.49	fenchol	0.03
15.59	chrysanthenone	0.01
15.74	cis-p-menth-2-en-1-ol	0.02
15.85	α-campholenal	0.01
16.47	E-pinocarveol	0.02
**16.82**	**camphor**	**11.53**
**17.99**	**endo-borneol**	**3.25**
18.39	4-terpineol	0.72
18.70	p-cymen-8-ol	0.02
**19.09**	**α-terpineol**	**1.71**
19.58	verbenone	0.13
22.70	E-ascaridol glycol	0.02
**23.17**	**bornyl acetate**	**1.03**
24.79	unknown	0.03
25.90	α-cubebene	0.04
26.83	α-ylangene	0.05
27.11	α-copaene	0.18
**28.94**	**β-caryophyllene**	**4.48**
29.36	β-copaene	0.04
29.69	alloaromadendrene	0.03
30.41	α-caryophyllene	0.38
31.24	γ-muurolene	0.11
32.20	cadinadiene	0.03
32.63	β-bisabolene	0.03
32.76	γ-cadinene	0.06
32.98	β-cadinene	0.15
35.38	epoxycaryophyllene	0.21

[1] retention time (rt)

[2] Percentage of each compound according to the total chromatogram area.

Table S5. *Essential oil composition of Tagetes minuta identified by GC-MS.*

rt (min)[1]	compound	% TIC[2]
7.07	α-pinene	0.06
7.51	propyl 2-methylbutyrate	0.08
7.72	camphene	0.08
8.65	sabinene	0.33
8.84	β-pinene	0.05
10.92	p-cymene	0.08
**11.13**	**limonene**	**5.26**
**11.50**	**Z-β-ocimene**	**41.95**
11.97	E-β-ocimene	0.28
**12.23**	**2,6-Dimethyloct-7-en-4-one (dihydrotagetone)**	**21.15**
13.12	1-octanol	0.07
13.28	α-pinene epoxide	0.37
14.11	3-methyl-2-(2-methyl-2-butenyl)-furan (rosefuran)	0.07
14.29	monoterpene epoxide	0.16
14.49	linalool	0.08
15.12	allyl (2E)-2-methyl-2-butenoate	0.11
15.83	(4E,6Z)-2,6-dimethyl-2,4,6-octatriene (neo-alloocimene)	0.60
15.91	E-β-ocimene epoxide (myroxide)	0.40
16.37	(E,E)-2,6-dimethyl-2,4,6-octatriene (E,E-alloocimene)	0.39
16.65	(5E)-2,6-dimethyl-5,7-octadien-4-one (E-tagetone)	0.22
**16.96**	**(5Z)-2,6-dimethyl-5,7-octadien-4-one (Z-tagetone)**	**1.07**
17.80	rosefuran epoxide	0.10
18.76	methyl salicylate	0.29
19.33	2,6-dimethylocta-3,5,7-trien-2-ol isomer	0.15
19.65	2,6-dimethylocta-3,5,7-trien-2-ol isomer	0.17
19.80	octyl acetate	0.16
20.65	(Z)-2,6-dimethylocta-2,5,7-trien-4-one (Z-tagetenone)	0.16
21.62	unknown	0.33
21.86	1,4,4-trimethyl-8-oxabicyclo[5.1.0]oct-5-en-2-ol	0.11
**22.31**	**carvone**	**1.25**
22.39	unknown	0.53
24.62	(E,E)-2,4-decadienal	0.22
25.36	piperitenone	0.25
25.62	7-epi-silphiperfol-5-ene	0.14
26.86	unknown	0.15
27.26	modhephene	0.42
28.85	β-caryophyllene	0.51
30.34	α-caryophyllene	0.19
35.13	spathulenol	0.73
35.31	epoxycaryophyllene	0.16
42.70	unknown	0.24
46.69	unknown	0.74
47.43	ethyl palmitate	0.44
47.56	2-methyl-6-(4-methyl-5-(3-methylbut-2-enoyl)cyclohex-3-en-1-yl)hepta-2,5-dien-4-one isomer	0.86
**47.93**	**2-mthyl-6-(4-methyl-5-(3-methylbut-2-enoyl)cyclohex-3-en-1-yl)hepta-2,5-dien-4-one isomer**	**1.55**
48.09	unknown	0.48
**48.38**	**2,6-ditert-butyl-4-methylphenyl 2-methylcyclopropanecarboxylate**	**2.15**
**48.44**	**(Z)-2-methyl-6-(4-methyl-5-(3-methylbut-2-enoyl)cyclohex-3-en-1-yl)hepta-2,5-dien-4-one**	**1.29**

48.51	C20H28O2	0.95
**48.79**	**uknown**	**1.38**
**48.81**	**ethyl linolate**	**2.06**
**48.86**	**ethyl oleate**	**1.45**
**49.04**	**unknown**	**6.92**
49.14	unknown	0.59

[1] retention time (rt)

[2] Percentage of each compound according to the total chromatogram area.

Table S6. Essential oil composition of *Thymus zygis* identified by GC-MS.

rt (min)[1]	compound	% TIC[2]
6.77	5.5-dimethyl-1-vinylbicyclo[2.1.1]hexane	0.26
6.85	tricyclene	0.06
6.98	2-thujene	0.20
**7.26**	**α-pinene**	**3.82**
7.90	camphene	0.76
8.85	sabinene	0.70
9.04	β-pinene	0.30
9.16	1-octen-3-ol	0.06
9.39	3-octanone	0.09
**9.59**	**β-myrcene**	**4.69**
9.91	3-octanol	0.06
10.30	α-phellandrene	0.30
**10.79**	**α-terpinene**	**2.10**
**11.15**	**p-cymene**	**4.52**
**11.35**	**limonene**	**3.63**
11.40	β-phellandrene	0.47
11.48	eucalyptol	0.13
12.17	E-β-ocimene	0.02
**12.70**	**γ-terpinene**	**4.28**
**13.27**	**sabinene hydrate**	**1.87**
13.96	terpinolene	0.88
14.05	linalool oxide	0.34
14.18	dimethylstyrene	0.05
**14.89**	**linalool**	**48.36**
14.95	hotrienol	0.79
15.82	cis-p-menth-2-en-1-ol	0.46
16.54	E-pinocarveol	0.03
16.66	E-p-Menth-2-en-1-ol	0.33
16.84	camphor	0.29
17.34	2.6-dimethyl-1.5.7-octatrien-3-ol	0.05
**18.04**	**endo-borneol**	**2.10**
18.25	trans-pyranoid linalool oxide	0.08
**18.50**	**4-terpineol**	**11.09**
18.77	p-cymen-8-ol	0.14
**19.15**	**α-terpineol**	**1.81**
19.26	dihydrocarvone	0.19
19.55	E-dihydrocarvone	0.19
19.79	E-piperitol	0.17
20.24	E-carveol	0.07
20.51	nerol	0.05
20.65	bornyl formate	0.20
21.16	2-Isopropyl-1-methoxy-4-methylbenzene	0.12
21.35	carvone	0.04
**21.73**	**linalyl acetate**	**1.11**
22.47	p-mentha-1.8-dien-3-one	0.08
22.75	1.4-dihydroxy-p-menth-2-ene	0.11
23.21	bornyl acetate	0.14
23.52	Carvacrol	0.70
23.85	thymol	0.10

24.84	unknown	0.08
27.48	β-bourbonene	0.05
**28.97**	**β-caryophyllene**	**1.06**
32.06	elixene	0.06
33	cadinene isomer	0.05
35.21	spathunelol	0.10
35.40	epoxycaryophyllene	0.19

[1] retention time (rt)

[2] Percentage of each compound according to the total chromatogram area.

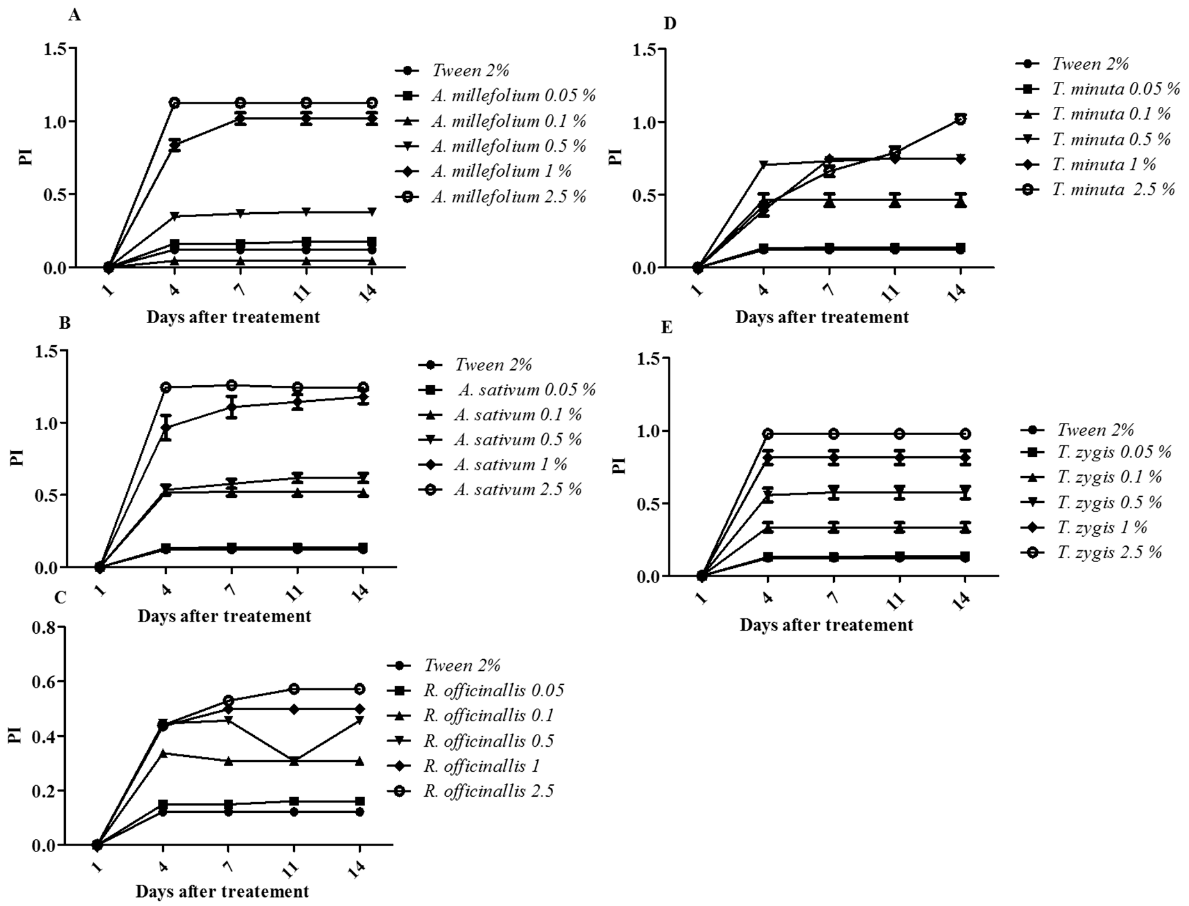

Figure S1. The phytotoxicity index (mean ± SE) of the essential oils of *Achillea millefolium* (A). *Allium sativum* (B). *Rosmarinus officinalis* (C). *Tagetes minuta* (D). and *Thymus zygis* (E) on tomato growth after 14 days of treatment and compared to the control (Tween 2%).
